# Supplementary material for: Socio-ecological impacts of the 2025 Los Angeles urban fires on communities, neighborhoods, and homes
Source: Nat Commun. 2026 Apr 30;17:3941. doi: 10.1038/s41467-026-71376-1 (PMC13133365; doi:10.1038/s41467-026-71376-1)
Supplement: Supplementary file 1 — Supplementary Information [file 41467_2026_71376_MOESM1_ESM.docx]

**Supplementary Information**

Socio-Ecological Impacts of the 2025 Los Angeles Urban Fires on Communities, Neighborhoods, and Homes

Carl A. Norlen^1,2*^, Sadikshya Sharma^1^, Francisco J. Escobedo^1^

^1^U.S. Department of Agriculture, Forest Service, Pacific Southwest Research Station, Riverside, CA

^2^U.S. Geological Survey, National Land Imaging Program, Reston, VA

*Corresponding author emails: [cnorlen@usgs.gov](mailto:cnorlen@usgs.gov), [canorlen@gmail.com](mailto:canorlen@gmail.com)

**List of Supplementary Items**

Supplementary Methods ............................................................................................... Pages 3 to 8

Supplementary Table 1. Community and neighborhood-scale socio-ecological variables used to analyze direct fire impacts in urban Eaton and Palisades...................................................... Page 9

Supplementary Table 2. Parcel-scale socio-ecological variables for analyses of direct fire impacts in the Eaton and Palisades, California study areas............................................................... Page 10

Supplementary Figure 1. Summary of direct fire impacts by structure category for the Eaton and Palisades fires....................................................................................................................... Page 11

Supplementary Figure 2. Counts of predicted race of property owners based on their names for the Eaton and Palisades Fires............................................................................................... Page 12

Supplementary Table 3. Neighborhood-Level Descriptive Summary of Eaton and Palisades Fires .............................................................................................................................................. Page 13

Supplementary Figure 3. The relationship between urban morphology and socio-demographic characteristics and direct fire impacts at the parcel-scale.................................................... Page 14

Supplementary Table 4. Summary of Parcel-Scale Variables in the Eaton and Palisades Fires .............................................................................................................................................. Page 15

Supplementary Figure 4. Map of fire perimeters from 2025 and pre-2025 for the areas affected by the Palisades fire............................................................................................................. Page 16

Supplementary Figure 5. Relationship between neighborhood-scale urban morphology characteristics and direct fire impacts.................................................................................. Page 17

Supplementary Figure 6. Relationship between neighborhood-scale socio-economic characteristics and direct fire impacts................................................................................. Page 18

Supplementary Table 5. Socio-ecological scale and variables and their importance in fire impact analyses for neighborhoods and homes in the Eaton and Palisades Fire............................. Page 19

Supplementary References........................................................................................ Pages 20 to 21

**Supplementary Methods**

Analyses of previous urban fires demonstrate that community, neighborhood, and parcel scales are all important in understanding their impacts ^1,2^. U.S. Census data ^3,4^ was used for community and neighborhood scale analysis of urban morphology and sociodemographic factors and Los Angeles County parcel data was used for parcel-scale analyses (Supplementary Table 1).

We joined DINS and LA County Parcel data using the Assessor’s Identification Number (AIN), Assessor’s Parcel Number (APN) and Core Logic data. We spatially joined the USACE structure inventory and Microsoft building footprint data to the LA County parcel data using a spatial join to the nearest feature. The Microsoft building footprint ^5^ data represents a static snapshot of building footprints for 2019-2020, so we assumed this is an accurate pre-fire representation of building footprint for Eaton and Palisades which ignores any buildings constructed or removed from 2021-2024. We believe that this is a reasonable assumption as no fires occurred within either community from 2021-2024 (Figure 1b).

*Urban morphology*

Defensible space buffers (DSB) are zones around homes in high fire hazard areas that regulate the amount and types of vegetation and other flammable fuels around homes and that are proposed to soon be enforced via local and state ordinances ^6^.

*Fire perimeter analysis*

We retrieved the 2025 Eaton and Palisades fire perimeters from National Interagency Fire Center (NIFC) ^7^ and other historical fire perimeter data through 2024 from CALFIRE’s Fire and Resource Assessment Program (FRAP) GIS Mapping and Data Analytics portal ^8^. A 100-m buffer was included in all affected census blocks that intersected the Eaton and Palisades fires. We then calculated the Proportional Fire Affected area (PFA) of each census block (see Eq. 1) by also using a spatial intersection analysis with the sf package ^9^ to calculate the fire affected area (FAA) in hectares burned. We then divided FAA by the census area (CA) or area of each census block or block group to calculate PFA using Equation 1:

$$\mathrm{PFA}=\frac{\mathrm{FAA}}{\mathrm{CA}} (Eq. 1)$$

*Urban tree cover*

We used a raster layer of 2022 urban tree cover produced by Earth Define from National Agriculture Imagery Program (NAIP) imagery. We retrieved the 0.6-m resolution image for Los Angeles, Anaheim and Long Beach ^10^. We used Google Earth Engine (GEE) ^11^ to retrieve the total number of pixels with tree cover and the total number of pixels within each US Census (2020) block. We then calculated the proportion of UTC in each census block by dividing the number pixels with tree cover by the total number of pixels to produce the ratio of urban tree cover to total land area ^12^.

*Structure footprint area and replacement value*

We calculated the density of structures or structure footprint area from the sum of all building footprints within a neighborhood or a parcel. We did so by calculating the area of all homes and structures then intersecting each urban U.S. census block or parcel (m^2^) with the Microsoft building footprint data ^5^ and then divided by the area of each U.S. census block or parcel (ha).

The home replacement value was obtained from the United States Army Corps of Engineer’s (USACE) National Structure Inventory (NSI) data ^13^. Median home replacement value ($) was obtained by taking the median of all structures from NSI data that intersected with each urban U.S. census block ^14^. The median replacement value of homes was used as a proxy for the relative real estate premium of homes across the affected communities. Replacement value is the U.S. dollar per square foot replacement value of the structure category, depreciated at 1% of replacement value for the first 20-years using 2021 price levels according to the USACE. After 20 years structures are assumed to retain 80% of their replacement value through routine maintenance.

*Structures in Defensible Space Buffer Zones*

To understand the influence of proximity to other homes on direct fire impacts at the neighborhood-scale, we calculated the mean number of structures in DSB Zones 0, 1, and 2 for each U.S. census block ^14^ (Supplementary Table 1). For the parcel-scale analysis we used individual building footprint within the Microsoft dataset ^5^ to calculate the number of structures within DSB Zones 0, 1, and 2 and calculated the number of structures and homes for each parcel. We then linked the output values to each U.S. census block by doing a spatial join with GEE ^11^ and calculated the mean number of structures in each DSB Zone within each U.S. census block.

*Direct fire impacts (structure/home destruction)*

We analyzed the degree of damage or destruction to individual homes by joining CALFIRE’s Damage Inspection (DINS) data with L.A. County parcel data ^15^ and filtering for structures within or adjacent to the Palisades and Eaton fire perimeters. For each U.S. census block, we calculated the total number of structures as well as the percentage of structures destroyed, damaged, or with no damage. We defined destroyed structures with the following DINS data categories: “Destroyed (>50%)”, “Affected (1-9%)”, “Minor (10-25%)”, or “Major (26-50%)” and “No Damage (0% damage)”. We calculated the destroyed portion (DP) as the number of structures destroyed (SD) divided by the total number of structures (TS) for each census block and block group using Equation 2:

$$\mathrm{DP}= \frac{\mathrm{DS}}{\mathrm{TS}} \left( Eq. 2 \right)$$

For community and neighborhood-scale analyses, we filtered for U.S. census blocks that had: a PFA greater than 0, number of structures greater than 0, the census block had a population greater than 0, and the census block was designated as urban (UR20 = U).

*Fire impacted homes*

We cleaned and pre-processed the DINS dataset by removing all structures with DINS fire impacts categorized as “inaccessible” and all observations with missing values (i.e., NAs) for other variables. We analyed the patterns of direct urban fire impacts on homes and their occupants (e.g., destruction or damage to residential structures) by combining a variety of datasets that provided information on the structures, damage to structures, urban morphology, and people living in the structures (Supplementary Table 2).

*Socio-demographic data*

As mentioned in our Study Design, Datasets, and Socio-Demographics Variables section, additional social and economic variables including income and poverty levels, educational attainment, and language proficiency (Supplementary Table 1), were also sourced from the U.S. census American Community Survey (ACS) five-year estimates ^4^ for 2019-2023 at the urban U.S. census block group level. The U.S. Census Bureau’s Advanced Search tool was employed to filter both the 2020 decennial census data and the ACS five-year estimates for Los Angeles County. The spatial and non-spatial datasets were then joined using GEOIDs, unique numeric identifiers assigned to census blocks and block groups, as the primary matching key. We do note the limitations in the ACS data (i.e., tradeoffs in spatial-temporal resolutions, higher marginal and standard errors), however this is the best available data and has been used in other similar studies ^16,17^.

Property owner race results are in-line with U.S. census data for Eaton, however results for Palisades were not consistent (Supplementary Figure 2), so property owner race data were not used in subsequent analyses. For Palisades, a large proportion of homeowners were predicted to be of “Black” or “Hispanic” ethnicity which did not correspond to U.S. Census data. For analysis, we converted the four predicted ethnicities to “White” and “non-White” (i.e., Hispanic, Black, Asian).

*Spatial disaggregation and fire exposure weighting*

U.S. census blocks are the smallest U.S. census geographic unit, typically corresponding to a city block (10 ha approx.) and provide most of the data from Supplementary Table 1. Supplementary Table 1 also includes block group data from larger aggregations of blocks (400 ha approx.) and are the smallest unit for ACS data estimates.

*Fire exposure weighting and sensitivity analysis*

We calculated fire-weighted populations (Vw) to adjust block-level population using the proportion of each U.S. census block affected by either fire using Yadav et al.’s approach ^17^. A sensitivity analysis assessed the representativeness of using fire-exposed, population-disaggregated data at the block level.

*Spatial disaggregation*

Specifically, ACS block group-level variables were disaggregated to the U.S. census block level using a population-weighted disaggregation method ^18^. The proportion of U.S. census block population to total U.S. census block group population was used as a weighting factor following Equation 3:

$\omega= \left( \frac{\mathrm{Pop}_{b}}{\mathrm{Pop}_{\mathrm{bg}}} \right) (Eq. 3)$

where, $\boldsymbol{\omega}$ is weight for the U.S. census block in the U.S. census block group, Pop_b_ is a population of the U.S. census block, Pop_bg_ is a population of the U.S. census block group.

Each U.S. census block group-level variable X_bg_ was then disaggregated to the U.S. census block scale by multiplying ω using equation 4:

$${X_{b}=X}_{bg}\times\omega(Eq. 4)$$

where X_b_ is an estimated U.S. census block value, X_bg_ is a U.S. census block group value from ACS, and $\omega$ is weight for each U.S. census block in the U.S. census block group. This spatial disaggregation methodology has two assumptions, first intra-block group homogeneity enables block-level estimates through population-based proportional scaling ^19^. Second, all variables of interest vary proportionally with changes in population.

This involved aggregating estimated values from U.S. census block back to U.S. census block group level and comparing them to the original U.S. census block group data. Analyses identified discrepancies in the representation of certain sociodemographic groups, with some groups being underrepresented in the block-level analysis. To address this, analytic weights derived from 2020 census block-scale population counts were applied during subsequent regression analysis ^20^. This approach accounts for variation in population size across U.S. census blocks and ensures that larger populations contribute proportionally more to estimated coefficients and helps prevent overrepresentation of sparsely populated U.S. census blocks. Analytic weights were implemented using Stata's *aweight* option in the regression command where each observation (i.e., U.S. census block) was weighed by its 2020 U.S. census population size (pop2020). Given these different sized analysis units and that fire impact data were available at the U.S. census block level, all variables were disaggregated to the U.S. census block level to serve as the subsequent unit of spatial analysis ^21,22^. This approach allowed the models to reflect effects relative to the total number of people instead of the total number of U.S. census blocks.

*Fire-weighted socio-demographic variables*

Calculating fire-weighted sociodemographic variables addresses the Modifiable Areal Unit Problem associated with overlaying fire perimeter polygons and different sized census blocks. The weighted sociodemographic variable (V_w_) was calculated by multiplying the sociodemographic variable of interest (V) by the proportion of fire-affected area (PFA) within each geographic unit, following Equation 5:

$V_{w}=V\times\mathrm{PFA} (Eq. 5)$

where, V_w_ represents the area-weighted sociodemographic variable for fire-affected (FA) census blocks. These weighted variables were used in subsequent analyses. Per capita income, unweighted and adjusted for 2023 inflation in US Dollars, was employed to directly represent income levels across U.S. census block groups.

*Statistical Analysis*

To investigate the relationship between fire-related damage and socio-ecological variables at the neighborhood-scale we used Generalized Linear Models (GLM) and Robust Regression techniques ^23,24^ to accommodate non-Gaussian error distributions and  to mitigate the influence of outliers while reducing risks associated with model misspecification. For subsequent parcel-scale analyses, binary logistic regression was used to model a binary outcome (i.e., home destruction) as a function of continuous predictors, given its suitability for non-normal error distributions and categorical responses ^25^.

To enable meaningful comparisons across variables measured using different scales, continuous independent variables were standardized through a z-score transformation ^26^. Additionally, the home destruction variable where >50% was fire damaged was coded as 1 (i.e., destroyed) and all other home damage categories (i.e., minor, moderate, and no damage) were coded as 0 to facilitate the use of logistic regression. Similarly, single-family residences were coded as 1 and all other residential types (e.g., multi-family, mobile homes) were coded as 0. Areas in the communities exposed to fire from 1910-2024 (i.e., Fire exposed 1910-2024) were also dummy coded using this approach.

Finally, we modeled the binary outcome of structure destruction (0 = intact, 1 = destroyed), by using a pooled logistic regression to better accounts for potential heterogeneity across different fire events, homes, and parcels ^24^. Correlation plots between the log odds of destruction and continuous predictors indicated non-linearity, so home destruction outcome was determined as a one-time event within a single fire incident i.e., (Eaton or Palisades). We performed all community and neighborhood-scale spatial analyses using the sf package ^9^ and GEE ^11^. All other statistical analyses were conducted using STATA software (version 19) and R (version 4.3.1) ^27,28^ and model diagnostics to evaluate model performance and fit included R^2^, pseudo-R², Akaike information criterion (AIC), and Bayesian Information Criterion (BIC).

**Supplementary Table 1.** Neighborhood-scale socio-ecological variables used to analyze direct fire impacts in the Eaton and Palisades communities. This table summarizes parcel- and neighborhood-scale socio-ecological variables and data sources used in the analysis. Values were used as inputs for descriptive and regression analyses. Table Notes: * indicates that all structures from Microsoft building footprints were included not just homes; ∗∗ indicates socio-demographic factors retrieved as ACS 5-year estimates were not available in Census 2020 summary files; ACS, American Community survey; USACE, United States Army Corps of Engineers; USDA FS, United States Department of Agriculture, Forest Service; CALFIRE, California Department of Forestry and Fire Protection; FRAP, Fire Resources Assessment Program; DINS, Damage Inspection Program; DSB, Defensible Space Buffer

| Variables | Description of the variables | Sources |
| --- | --- | --- |
| **Sociodemographic** |  |  |
| Population | Total affected population in census block | Census 2020 |
| Gender | % Population male and female | Census 2020 |
| Age distribution | % Population in different age groups | Census 2020 |
| Ethnicity | % Population Hispanic/not Hispanic | Census 2020 |
| Race | % Population under different races | Census 2020 |
| Housing occupancy | % Housing units occupied/vacant | Census 2020 |
| Housing tenure | % Housing units owner/renter occupied | Census 2020 |
| Language proficiency | % Population non-English speakers | ACS 5-year estimates** |
| Income | 2023 inflation adjusted Per capita Income ($) | ACS 5-year estimates** |
| Poverty | % Population below poverty level | ACS 5-year estimates** |
| Median structure replacement value ($) | Median structure replacement value for disaster planning and response purpose | USACE |
| Education | % Population under different levels of education | ACS 5-year estimates** |
| **Urban Morphology** |  |  |
| Pre-Fire urban tree cover | % of pre-fire urban tree cover (2022) | USDA FS Region 5 |
| Fire exposure 1910-2024 (%) | % block within 1910-2024 fire perimeters | CALFIRE FRAP |
| Structure footprint area (m^2^ ha^-1^)* | Area of structure footprints per area of land in the census block | Microsoft Building Footprints and US Census (2020) |
| Median Year Structure Built | The median year structures were built within the census block | LA Assessors Office |
| Mean Number of Structures in DSB 0* | Average number of structures within DSB Zone 0 (0-1.5 m) | Microsoft Building Footprints |
| Mean Number of Structures in DSB 0 & 1* | Average number of structures within DSB Zones 0 and 1 (0-9.1 m) | Microsoft Building Footprints |
| Mean Number of Structures in DSB 0, 1, & 2* | Average number of structures within DSB Zone 0, 1, and 2 (0-30.5 m) | Microsoft Building Footprints |
| Fire exposure 2025 (%) | % of the census block within 2025 fire perimeter | CALFIRE FRAP |
| **Home/Parcel Impacts** |  |  |
| Destroyed (%) | % of the structures within the census block that were destroyed according to CALFIRE | CALFIRE DINS |
| No Damage (%) | % of structures within the census block that had no damage according to CALIFRE | CALFIRE DINS |

| **Supplementary Table 2.** Parcel-scale socio-ecological variables for analyse s of direct fire impacts in the Eaton and Palisades communities. This table lists parcel-scale socio-ecological variables and data sources used in the analysis of direct fire impacts. No statistical analyses were conducted for this table. Table Notes: * all structures from Microsoft building footprints were included, not just homes; ACS, American Community survey; L.A., Los Angeles; USACE, United States Army Corps of Engineers; USDA FS, United States Department of Agriculture, Forest Service; CALFIRE, California Department of Forestry and Fire Protection; FRAP, Fire Resources Assessment Program; DINS, Damage Inspection Program; DSB, Defensible Space Buffer | | |  |
| --- | --- | --- | --- |
| Variables | Description of the variables | Sources |  |
| **Sociodemographic** |  |  |  |
| Total number of residents | The total number of residents of a home during the night hours | USACE National Structure Inventory |  |
| Proportion of residents above 65 | The proportion of the total residents over 65 years of age | USACE National Structure Inventory |  |
| Homeowner race | Predicted race of the homeowner based on name | CoreLogic and *rethnicity* model |  |
| Home Replacement Value ($) | | Median replacement value of the main structure for the property | USACE National Structure Inventory |
| **Urban Morphology** |  |  |  |
| Number of structures in DSB Zone 0* | Number of overlapping structures in DSB 0 (within 1.5 m) of the home | Microsoft Building footprints |  |
| Number of structures in DSB Zone 1* | Number of overlapping structures in DSB 1 (within 1.5-9.1 m) of the home | Microsoft Building footprints |  |
| Number of structures in DSB Zone 2* | Number of overlapping structures in DSB 2 (within 9.1-30.5 feet) of the home | Microsoft Building footprints |  |
| Home/Parcel Impacts |  |  |  |
| Type of home | Use of home for single family residential, multi-family residential, etc. | CALFIRE DINS |  |
| Year Home Built | Year that the home was built | L.A. County Parcel |  |
| Structure Footprint Area (m^2^ ha^-1^)* | The area of all structures per unit area for each parcel. | Microsoft Building footprints and LA County Parcels |  |
| 1910-2024 Fire exposure (Y/N) | Categorical indicator of whether a home was exposed to wildfire from 1910 – 2024 according to CALFIRE fire perimeters | CALFIRE FRAP |  |
| 2025 Fire impacts | Fire impact to homes from “No Damage” to “Destroyed (>50%) | CALFIRE DINS |  |


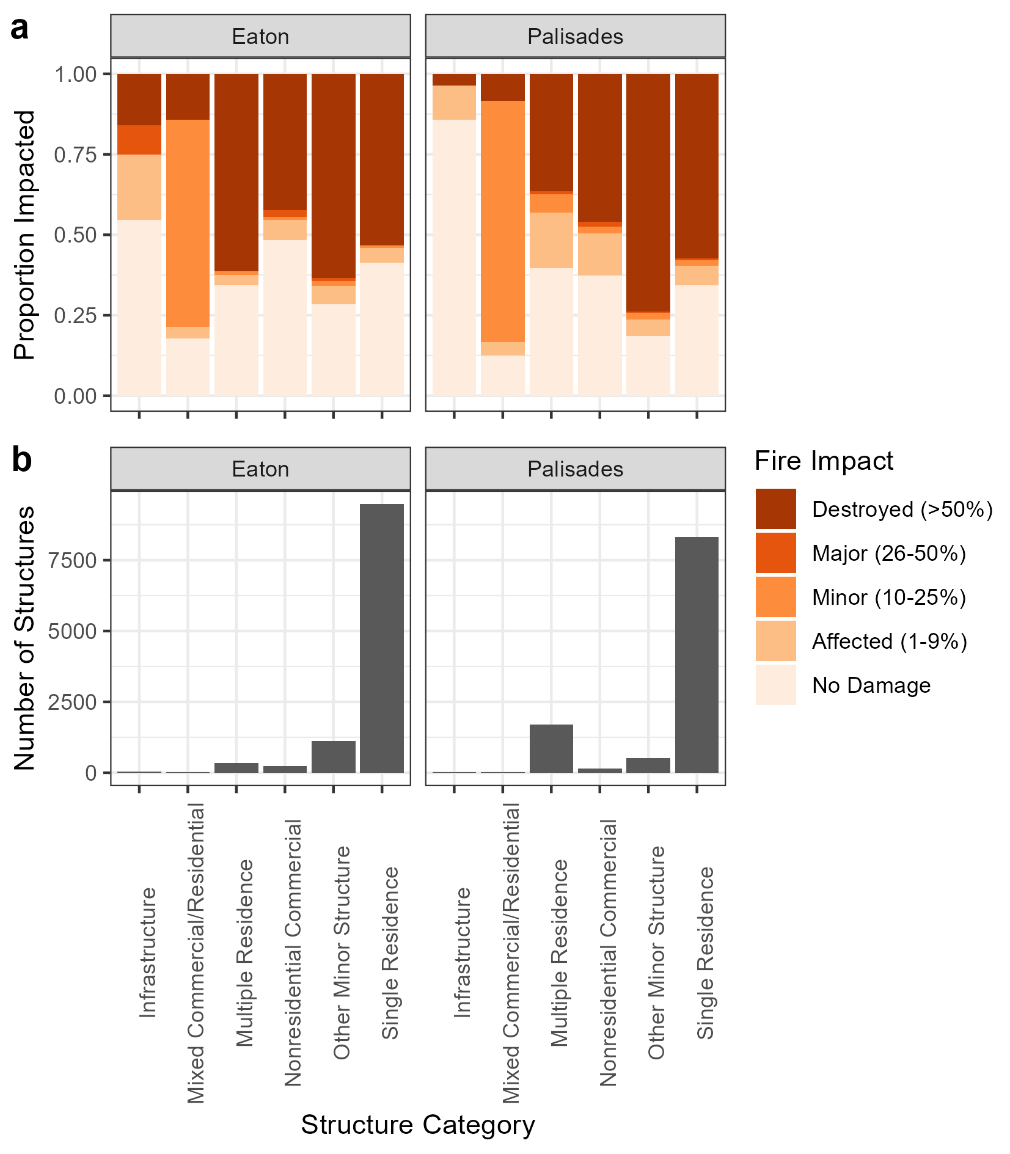


**Supplementary Figure 1.** Summary of direct fire impacts by structure category for the Eaton and Palisades communities. The two panels show (a) the proportion of damage and (b) the number of structures separated by structure category. The colors in panel a represent the level of damage to homes.


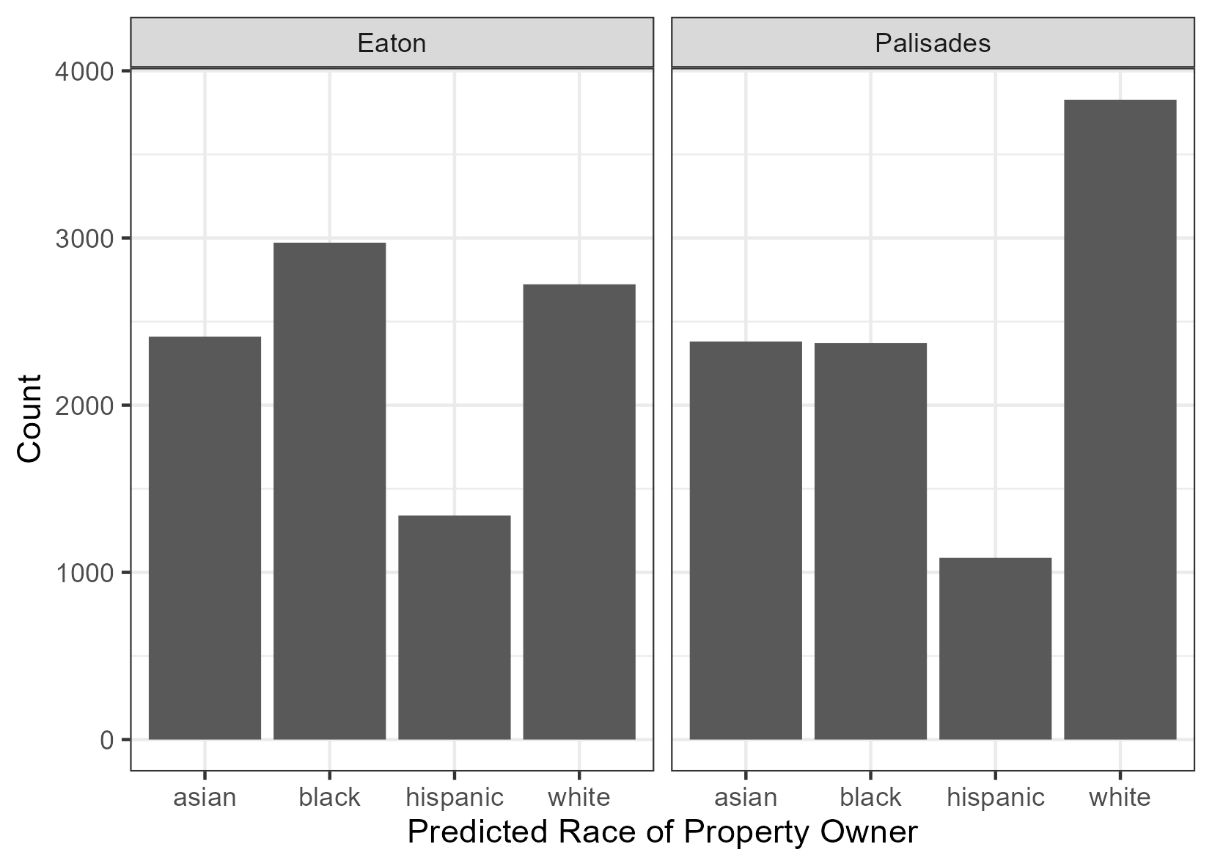


**Supplementary Figure 2.** Counts of predicted race of property owners based on their first and last names for the communities impacted by the Eaton and Palisades fires.

| **Supplementary Table 3.** Neighborhood-scale Descriptive Summary of Eaton and Palisades Fires. Values are neighborhood-scale descriptive statistics summarized as means and standard deviations. No hypothesis testing was conducted for this table. Table Notes: * includes all structures from Microsoft building footprints, not just homes; DSB, defensible space buffer | | | | | |
| --- | --- | --- | --- | --- | --- |
|  |  | Eaton Fire | | Palisades Fire | |
| Variables | Description of Variables | Mean | Std. dev. | Mean | Std. dev. |
| Population | Total exposed block population | 168.17 | 108.85 | 271.64 | 268.90 |
| Fire affected population | Population affected by 2025 fire | 149.29 | 99.90 | 257.00 | 261.26 |
| Block area | Area of census block (ha) | 13.04 | 24.75 | 24.68 | 32.63 |
| 2025 Fire exposure (%) | % US Census block exposed to fire in 2025 | 90.00 | 19.64 | 94.94 | 14.09 |
| Homes destroyed by fire (%) | Proportion of homes destroyed by 2025 fire (%) | 69.69 | 30.06 | 59.70 | 34.19 |
| 1910-2024 Fire exposure (%) | % US Census block exposed to fire from 1910-2024 | 17.80 | 34.48 | 46.85 | 46.53 |
| DSB Zone 0* | Mean number of structures in DSB Zone 0 | 0.20 | 0.12 | 0.18 | 0.15 |
| DSB Zone 1* | Mean number of structures in DSB Zone 1 | 1.95 | 0.52 | 1.79 | 0.53 |
| DSB Zone 2* | Mean number of structures in DSB Zone 2 | 6.76 | 1.95 | 6.66 | 2.57 |
| Structure replacement value ($) | Estimate cost to replace structure ($) | 249073.00 | 78921.09 | 348719.70 | 74531.60 |
| Structure footprint area* (m^2^ ha^-1^) | Area of structures (m^2^) per area of land surface (ha) | 1743.64 | 577.15 | 1937.93 | 863.05 |
| Median Year Home Built | Median year homes were built in the census block | 1945.83 | 16.48 | 1963.18 | 15.19 |
| Home built after 2008 (%) | Proportion of homes built after 2008 | 0.57 | 3.21 | 5.77 | 6.92 |
| Pre-Fire Tree Cover (%) | Pre-Fire Tree Cover (%) | 29.64 | 9.03 | 23.87 | 9.55 |
| Gender | Male (%) | 47.59 | 9.29 | 47.72 | 10.57 |
|  | Female (%) | 52.41 | 9.29 | 52.28 | 10.57 |
| Age | Below 5 years (%) | 3.97 | 2.86 | 3.52 | 3.11 |
|  | 5-19 years (%) | 15.57 | 7.03 | 16.76 | 8.60 |
|  | 20-64 years (%) | 58.51 | 11.01 | 51.07 | 9.77 |
|  | 65 and over (%) | 21.67 | 11.17 | 25.69 | 9.78 |
| Ethnicity | Hispanic (%) | 26.16 | 16.71 | 6.07 | 4.58 |
|  | Not Hispanic (%) | 73.84 | 16.71 | 93.93 | 4.58 |
| Race | White (%) | 45.53 | 17.07 | 82.53 | 7.78 |
|  | African American (%) | 19.06 | 11.61 | 0.66 | 1.53 |
|  | American Indian / Alaska Native (%) | 0.01 | 0.02 | 0.00 | 0.01 |
|  | Asian (%) | 8.07 | 7.35 | 6.46 | 4.73 |
|  | Native Hawaiian / Pacific Islander (%) | 0.12 | 0.48 | 0.10 | 0.49 |
|  | Other race (%) | 27.21 | 15.04 | 10.25 | 5.96 |
| Occupancy | Occupied structures (%) | 96.38 | 8.16 | 91.25 | 11.85 |
|  | Vacant structures (%) | 3.62 | 8.16 | 8.75 | 11.85 |
| Tenure | Owner occupied structures (%) | 76.60 | 26.74 | 79.28 | 24.59 |
|  | Renter occupied structures (%) | 23.40 | 26.74 | 20.56 | 24.39 |
| Language proficiency | English speakers (%) | 75.93 | 18.20 | 84.36 | 7.32 |
|  | Non-English speakers (%) | 24.07 | 18.20 | 15.64 | 7.32 |
| Education | No formal education (%) | 1.52 | 3.21 | 0.22 | 0.44 |
|  | High school (%) | 13.48 | 9.14 | 5.45 | 3.04 |
|  | Associate degree (%) | 8.84 | 7.32 | 3.96 | 2.56 |
|  | Bachelor’s degree (%) | 27.31 | 10.92 | 37.68 | 7.92 |
|  | Graduate/Professional degree (%) | 27.24 | 11.07 | 40.85 | 9.25 |
| Poverty | Below poverty (%) | 6.05 | 5.49 | 5.38 | 4.35 |
| Income | Per capita income | 70252.54 | 26227.94 | 140932.70 | 33485.22 |


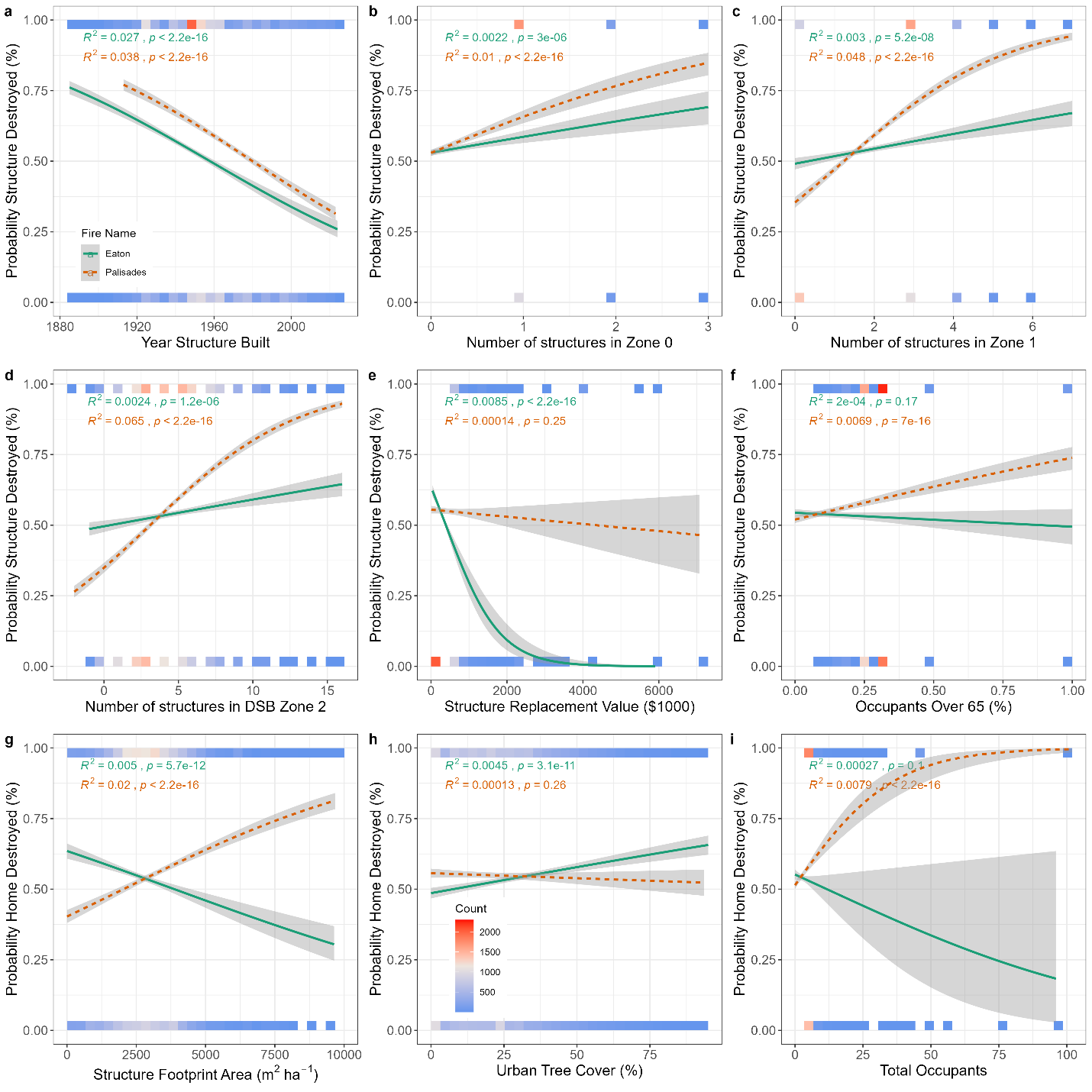


**Supplementary Figure 3.** The relationship between urban morphology (a, b, c, d, e, g, h) and socio-demographic (f, i) characteristics and direct fire impacts at the parcel-scale for both the Eaton (green) and Palisades (orange) communities. Lines represent fits for logistic regression models with the shaded regions representing standard errors. The colors from blue to red with cream as the midpoint represent the number of data points for the predictor variables.

| **Supplementary Table 4.** Summary of Parcel-scale Variables in the Eaton and Palisades communities. Values are parcel-scale descriptive statistics summarized as means and standard deviations for the Eaton and Palisades Fires. No hypothesis testing was conducted for this table. Table Notes: * includes all structures from Microsoft building footprints not just homes; DSB, Defensible Space Buffer | | | | | |
| --- | --- | --- | --- | --- | --- |
|  | Eaton Fire | | Palisades Fire | |  |
| Variables | Mean | Std. dev. | Mean | Std. dev. |  |
| Occupants over 65 yrs (%) | 0.12 | 0.14 | 0.13 | 0.18 |  |
| Total occupants | 2.80 | 2.10 | 2.87 | 4.87 |  |
| Predicted property owner race (white / non-white) | 0.28 | 0.45 | 0.39 | 0.49 |  |
| Home replacement value ($) | 280076.50 | 190150.60 | 442051.50 | 479711.80 |  |
| Pre-fire urban tree cover (%) | 28.85 | 18.06 | 20.74 | 15.81 |  |
| Number of structures in DSB Zone 0* | 0.18 | 0.42 | 0.16 | 0.40 |  |
| Number of structures in DSB Zone 1* | 1.87 | 1.04 | 1.68 | 0.97 |  |
| Number of structures in DSB Zone 2* | 4.65 | 2.59 | 4.24 | 2.79 |  |
| Structure footprint area* (m^2^ ha^-1^) | 2618.30 | 989.36 | 3120.71 | 1511.18 |  |
| 1910-2024 Fire exposure (Y / N) | 0.19 | 0.39 | 0.51 | 0.50 |  |
| Year home built | 1945.95 | 47.40 | 1961.33 | 118.64 |  |
| Single family homes | 0.86 | 0.35 | 0.79 | 0.41 |  |


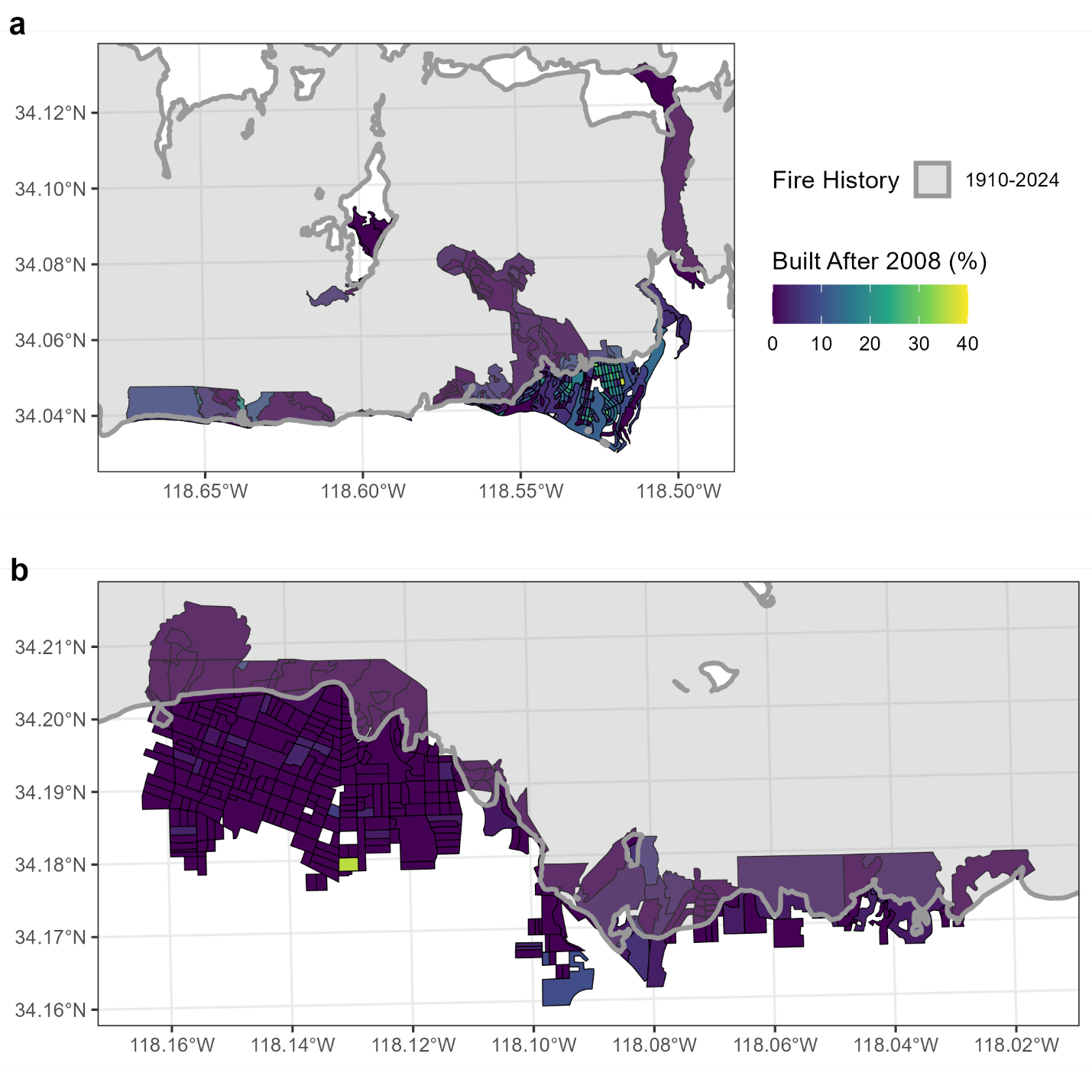


**Supplementary Figure 4.** Map showing the proportion of structures built after 2008 in the Palisades (a) and Eaton (b) communities. The map shows U.S. census blocks (black) as well as areas with 1910 to 2024 fire exposure (gray) for each community. The colors within each U.S. Census (2020) block represent the proportion of structures built after 2008 with dark blue representing low values (0%), green representing the mid-point (20%) and yellow representing the greatest values (40%).


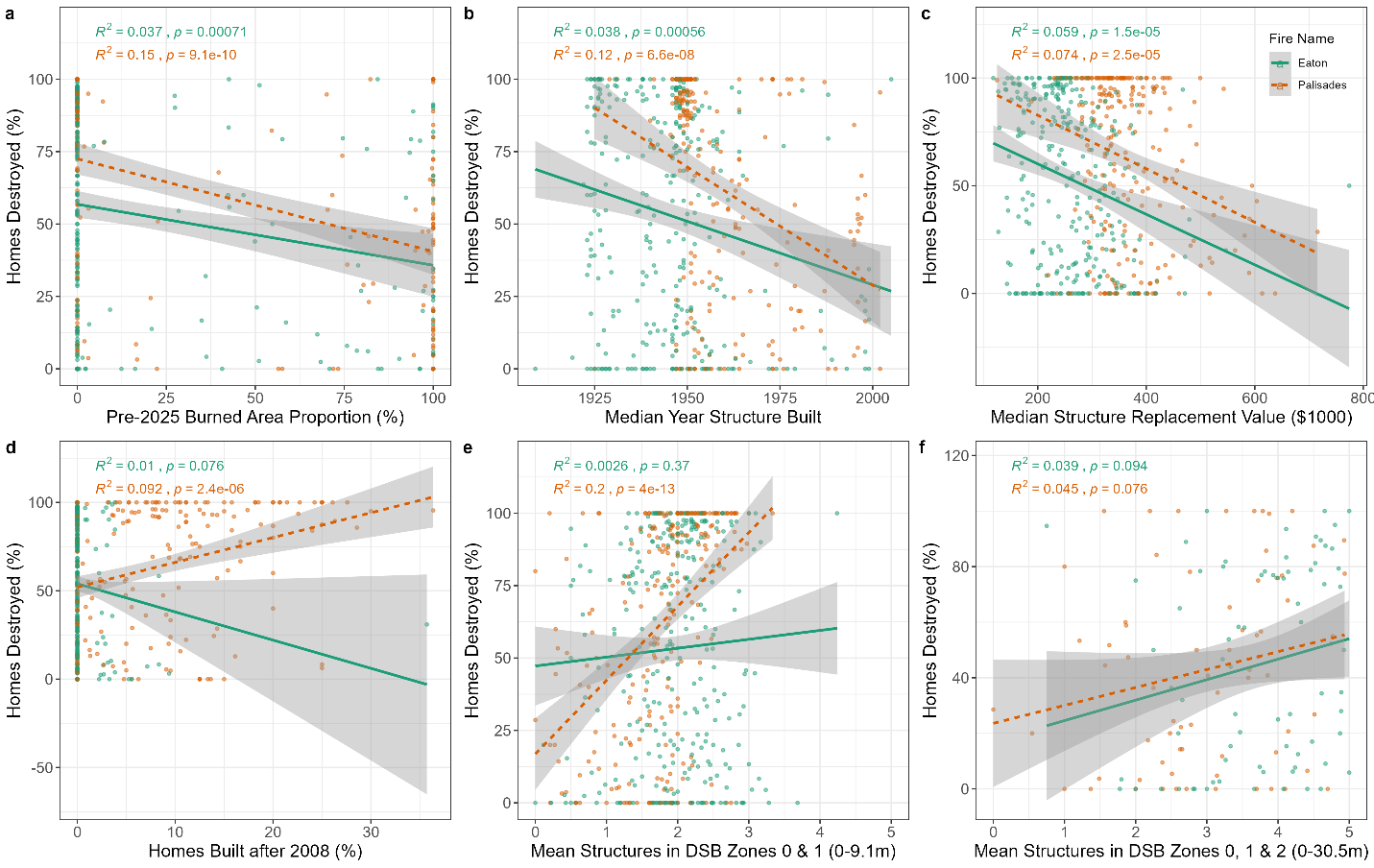


**Supplementary Figure 5.** Relationship between neighborhood-scale urban morphology characteristics (a, b, c, d, e, f) and direct fire impacts for the communities impacted by the Eaton (green) and Palisades (orange) fires. Fire impacts are measured in % homes destroyed in each U.S. census block. Lines show ordinary least squares linear model fits with the shaded regions representing standard errors.


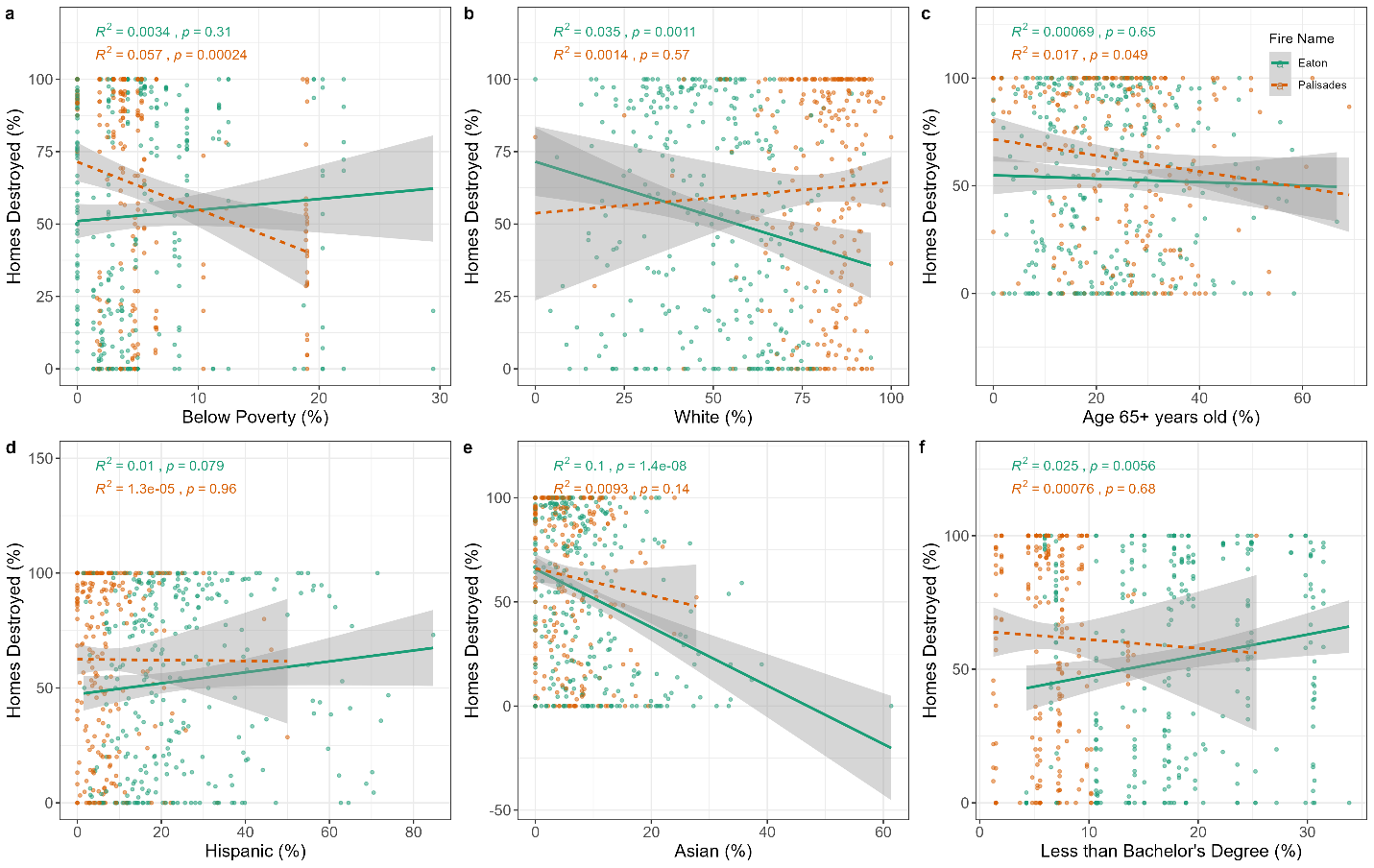


**Supplementary Figure 6.** Relationship between neighborhood-scale socio-economic characteristics (a, b, c, d, e, f) and direct fire impacts for the communities impacted by the Eaton (green) and Palisades (orange) fires. Fire impacts are measured in % homes destroyed in each U.S. census block. Lines show ordinary least squares linear regression fits with the shaded regions representing standard errors.

| **Supplementary Table 5.** Socio-ecological scale, variables and their importance in fire impact analyses at the neighborhood- and parcel-scale in the communities impacted by Eaton and Palisades fires. Table Notes: DSB, Defensible Space Buffer | | |
| --- | --- | --- |
| **Comparison** | **Correlates Similar** | **Correlates Different** |
| Palisades versus Eaton at Neighborhood-cale | Vacant (%), Renter (%), non-English speakers (%) | DSB, Year home built, Home replacement value, Structure footprint area, Homes built after 2008, Education level |
| Palisades versus Eaton at Parcel-scale | DSB, Home replacement value, Year home built | Total number occupants, Pre-fire tree cover, Single family homes |
| Neighborhood- versus Parcel-scale for Eaton | Pre-fire tree cover, 1910-2024 fire exposure, People over 65 years, Home replacement value | Structure footprint area |
| Neighborhood- versus Parcel-scale for Palisades | DSB, Pre-fire tree cover, Year home built, 1910-2024 fire exposure, People over 65 years |  |

**Supplementary References**

1. Metz, A. J., Fischer, E. C. & Liel, A. B. The Influence of Housing, Parcel, and Neighborhood Characteristics on Housing Survival in the Marshall Fire. *Fire Technol.* **60**, 4065–4097 (2024).

2. Escobedo, F. J., Yadav, K., Cappelluti, O. & Johnson, N. Exploring urban vegetation type and defensible space’s role in building loss during wildfire-driven events in California. *Landsc. Urban Plan.* **262**, 105421 (2025).

3. US Census Bureau. Census Demography and Housing Characteristics and Summary Files 1, 2, 3. (2021).

4. US Census Bureau. American Community Survey 5-year Estimates Detailed Tables. (2023).

5. Microsoft. US Building Footprints. (2020).

6. Penman, S. H., Price, O. F., Penman, T. D. & Bradstock, R. A. The role of defensible space on the likelihood of house impact from wildfires in forested landscapes of south eastern Australia. *Int. J. Wildland Fire* **28**, 4–14 (2018).

7. National Interagency Fire Center. WFIS Interagency Fire Perimeters to Date. (2025).

8. FRAP. 2023 Fire Perimeters. (2024).

9. Pebesma, E. Simple Features for R: Standardized Support for Spatial Vector Data. *R J.* **10**, 439 (2018).

10. Earth Define. US Tree Map: Seamless high-resolution tree canopy data. (2025).

11. Gorelick, N. *et al.* Google Earth Engine: Planetary-scale geospatial analysis for everyone. *Remote Sens. Environ.* **202**, 18–27 (2017).

12. Jensen, J. R. *Remote Sensing of the Environment : An Earth Resource Perspective*. (Upper Saddle River, NJ : Pearson Prentice Hall, 2007).

13. USACE. National Structure Inventory. (2022).

14. U.S. Census Bureau. 2020 U.S. Census block boundaries. (2021).

15. LA County. 2025 Parcels with DINS data. (2025).

16. Davies, I. P., Haugo, R. D., Robertson, J. C. & Levin, P. S. The unequal vulnerability of communities of color to wildfire. *PLOS ONE* **13**, e0205825 (2018).

17. Yadav, K., Escobedo, F. J., Thomas, A. S. & Johnson, N. G. Increasing wildfires and changing sociodemographics in communities across California, USA. *Int. J. Disaster Risk Reduct.* **98**, 104065 (2023).

18. Hallisey, E. *et al.* Transforming geographic scale: a comparison of combined population and areal weighting to other interpolation methods. *Int. J. Health Geogr.* **16**, 29 (2017).

19. Matsunaga, M., Ishikawa, K. M., Siriwardhana, C., Ahn, H. J. & Chen, J. J. Stepwise Proportional Weighting Algorithm for Single-Race Population Estimation Using Hawai‘i Census Data. *Hawaii J. Health Soc. Welf.* **82**, 97–103 (2023).

20. Coffman, D. L., Zhou, H., Castellano, K. E., Schuler, M. S. & McCaffrey, D. F. Sampling weighting strategies in causal mediation analysis. *BMC Med. Res. Methodol.* **24**, 133 (2024).

21. Gigli, K. H. Data Disaggregation: A Research Tool to Identify Health Inequities. *J. Pediatr. Health Care* **35**, 332–336 (2021).

22. Patil, S., Pflugradt, N., Weinand, J. M., Stolten, D. & Kropp, J. A systematic review of spatial disaggregation methods for climate action planning. *Energy AI* **17**, 100386 (2024).

23. Hossain, M. R. & Smirnov, O. Analyzing the risk factors of residential fires in urban and rural census tracts of Ohio using panel data analysis. *Appl. Geogr.* **151**, 102863 (2023).

24. Jennings, C. R. Social and economic characteristics as determinants of residential fire risk in urban neighborhoods: A review of the literature. *Fire Saf. J.* **62**, 13–19 (2013).

25. Kupek, E. Beyond logistic regression: structural equations modelling for binary variables and its application to investigating unobserved confounders. *BMC Med. Res. Methodol.* **6**, 13 (2006).

26. Gelman, A. & Hill, J. *Data Analysis Using Regression and Multilevel/Hierarchical Models*. (Cambridge University Press, 2007).

27. StataCorp. Stata Statistical Software. StataCorp, LLC (2025).

28. R Core Team. R: A language and environment for statistical computing. R Foundation for Statistical Computing (2020).
