# Supplementary material for: Socio-ecological impacts of the 2025 Los Angeles urban fires on communities, neighborhoods, and homes
Source: Nat Commun. 2026 Apr 30;17:3941. doi: 10.1038/s41467-026-71376-1 (PMC13133365; doi:10.1038/s41467-026-71376-1)
Supplement: Supplementary file 2 — Reporting Summary [file 41467_2026_71376_MOESM2_ESM.pdf]

nature portfolio

Corresponding author(s): Carl A. Norlen

Last updated by author(s): 02/18/2026

Reporting Summary

Nature Portfolio wishes to improve the reproducibility of the work that we publish. This form provides structure for consistency and transparency in reporting. For further information on Nature Portfolio policies, see our Editorial Policies and the Editorial Policy Checklist.

Statistics

For all statistical analyses, confirm that the following items are present in the figure legend, table legend, main text, or Methods section.

n/a	Confirmed	
[ ]	[x]	The exact sample size (*n*) for each experimental group/condition, given as a discrete number and unit of measurement
[x]	[ ]	A statement on whether measurements were taken from distinct samples or whether the same sample was measured repeatedly
[ ]	[x]	The statistical test(s) used AND whether they are one- or two-sided<br>*Only common tests should be described solely by name; describe more complex techniques in the Methods section.*
[ ]	[x]	A description of all covariates tested
[ ]	[x]	A description of any assumptions or corrections, such as tests of normality and adjustment for multiple comparisons
[ ]	[x]	A full description of the statistical parameters including central tendency (e.g. means) or other basic estimates (e.g. regression coefficient) AND variation (e.g. standard deviation) or associated estimates of uncertainty (e.g. confidence intervals)
[ ]	[x]	For null hypothesis testing, the test statistic (e.g. *F*, *t*, *r*) with confidence intervals, effect sizes, degrees of freedom and *P* value noted<br>*Give P values as exact values whenever suitable.*
[ ]	[x]	For Bayesian analysis, information on the choice of priors and Markov chain Monte Carlo settings
[x]	[ ]	For hierarchical and complex designs, identification of the appropriate level for tests and full reporting of outcomes
[ ]	[x]	Estimates of effect sizes (e.g. Cohen's *d*, Pearson's *r*), indicating how they were calculated

Our web collection on statistics for biologists contains articles on many of the points above.

Software and code

Policy information about availability of computer code

Data collection	We processed existing datasets from from USFS (Earth Define), CAL FIRE, LA County / Core Logic, Microsoft, the U.S. Census Bureau and US Army Corps of Engineers or further analysis using R 4.5.0, STATA, and Google Earth Engine. We also used Landsat 9 surface reflectance from USGS for as a map background in figure creation.
Data analysis	We used R 4.5.0, STATA version 19 and the Google Earth Engine Code Editor to do all analysis.

For manuscripts utilizing custom algorithms or software that are central to the research but not yet described in published literature, software must be made available to editors and reviewers. We strongly encourage code deposition in a community repository (e.g. GitHub). See the Nature Portfolio guidelines for submitting code & software for further information.

Data

Policy information about availability of data

All manuscripts must include a data availability statement. This statement should provide the following information, where applicable:

- Accession codes, unique identifiers, or web links for publicly available datasets
- A description of any restrictions on data availability
- For clinical datasets or third party data, please ensure that the statement adheres to our policy

All code for data cleaning, analysis, and figure generation as well as processed data for this study are available in the following figshare repository: https://doi.org/10.6084/m9.figshare.29936876. All code is also available in the following GitHub repository: https://github.com/carlnorlen/la-urban-fires.

Research involving human participants, their data, or biological material

Policy information about studies with human participants or human data. See also policy information about sex, gender (identity/presentation), and sexual orientation and race, ethnicity and racism.

Reporting on sex and gender	N/A
Reporting on race, ethnicity, or other socially relevant groupings	N/A
Population characteristics	N/A
Recruitment	N/A
Ethics oversight	N/A

Note that full information on the approval of the study protocol must also be provided in the manuscript.

Field-specific reporting

Please select the one below that is the best fit for your research. If you are not sure, read the appropriate sections before making your selection.

☐ Life sciences ☐ Behavioural & social sciences ☒ Ecological, evolutionary & environmental sciences

For a reference copy of the document with all sections, see nature.com/documents/nr-reporting-summary-flat.pdf

Ecological, evolutionary & environmental sciences study design

All studies must disclose on these points even when the disclosure is negative.

Study description	We studied the impacts of the 2025 LA Fires on urban communities at the neighborhood and residential parcel scales
Research sample	We focused our analysis on U.S. Census Blocks impacted by the 2025 Eaton and Palisades Fires to understand their impacts on communities at two spatial scales (residential parcels and neighborhoods).
Sampling strategy	N/A
Data collection	N/A
Timing and spatial scale	N/A
Data exclusions	We filtered blocks and parcels from the analysis that had missing. How we filtered the data for missing information is described in more detail in the Main Text and Supplementary Information of the manuscript.
Reproducibility	We describe all analysis and data collection in detail in the manuscript and will make all code and processed data used in the creation of figures and tables available in public repositories upon final acceptance of the manuscript.
Randomization	We analyzed the impacts of the Eaton and Palisades Fires separately from each other.
Blinding	N/A

Did the study involve field work? ☐ Yes ☒ No

Reporting for specific materials, systems and methods

We require information from authors about some types of materials, experimental systems and methods used in many studies. Here, indicate whether each material, system or method listed is relevant to your study. If you are not sure if a list item applies to your research, read the appropriate section before selecting a response.

Materials & experimental systems

n/a	Involved in the study
☒	☐ Antibodies
☒	☐ Eukaryotic cell lines
☒	☐ Palaeontology and archaeology
☒	☐ Animals and other organisms
☒	☐ Clinical data
☒	☐ Dual use research of concern
☒	☐ Plants

Methods

n/a	Involved in the study
☒	☐ ChIP-seq
☒	☐ Flow cytometry
☒	☐ MRI-based neuroimaging

Plants

Seed stocks	N/A
Novel plant genotypes	N/A
Authentication	N/A
